# Effect of PhenylEthanol Glycosides from *Cistanche Tubulosa* on Autophagy and Apoptosis in H22 Tumor-Bearing Mice

**DOI:** 10.1155/2022/3993445

**Published:** 2022-12-09

**Authors:** Xinxin Qi, Xiaotian Hou, Deqi Su, Zhuanxia He, Jun Zhao, Tao Liu

**Affiliations:** ^1^Department of Public Health, Xinjiang Medical University, Xinjiang Uyghur Autonomous Region, Urumqi 830054, Xinjiang, China; ^2^Medical Administration Division, Cancer Hospital Affiliated to Xinjiang Medical University, Xinjiang Uyghur Autonomous Region, Urumqi 830054, Xinjiang, China; ^3^Key Laboratory for Uighur Medicine, Institute of Medica of Xinjiang, Xinjiang Uyghur Autonomous Region, Urumqi 830054, Xinjiang, China

## Abstract

An effectual remedy for hepatocellular carcinoma (HCC) and knowledge of the mechanism are urgently needed. Researchers have found that CPhGs, an extract from *Cistanche tubulosa* (Schenk) Wight, had better antitumor effects, but its mechanism is still unknown. In the present study, using an H22 tumor-bearing mouse as a model, we investigated the antitumor effects of CPhGs and the effect of CPhGs on autophagy and apoptosis. Besides, we also discussed the role of autophagy with the help of HCQ and rapamycin. Our results show that CPhGs inhibit tumor growth and induce apoptosis and autophagy of tumor tissue. TUNEL staining displayed that tumor apoptosis rate increased after the intervention of CPhGs, and immunohistochemistry and western blot showed that cleaved-PARP and cleaved-caspase 3 were upregulated after the intervention of CPhGs, and these results were most pronounced in the high-dose group. Autophagy results revealed that CPhGs increased the number of autophagosomes, increased the level of LC3B-II, and decreased the level of p62. Finally, our results showed that excessive autophagy suppresses tumor growth, whereas inhibition of autophagy does the opposite, which indicated that CPhGs induced autophagic death in H22 hepatoma-bearing mice. These data altogether confirmed the involvement of apoptosis and autophagy in CPhGs treatment for HCC.

## 1. Introduction

Primary liver cancer is a catastrophic event because it is fourth in incidence and second in fatalities in China, accounting for 45% of global deaths and its low curative rate and high recurrence rate [1]. Currently, liver cancer has standard treatments, but there are many problems, including adverse effects, drug resistance, poor prognosis, and short survival [2, 3]. In recent studies, researchers have verified that some natural compounds have the potential effects to prevent and cure cancer [2]. Mounting reports have pointed out that Chinese medicine is not easy to produce drug resistance and has the advantage of prolonging the survival of tumor patients, effectively improving the patient's symptoms [4].

It was found that phenylethanoid glycosides (PhGs), a bioactive component from the fleshy stem of *Cistanche tubulosa* (Schenk) wight contains, play an essential role in anticancer. It has been shown that CPhGs could improve the hypoxic tumor microenvironment and enhance the effects of oxaliplatin via the HIF-*α* signaling pathway [5]. Echinacoside can effectively treat breast cancer by regulating Wnt/*β*-catenin signaling [6]. Nevertheless, the specific mechanisms underlying the antitumor effects of CPhGs remain poorly understood.

As we all know, the mechanisms of autophagy and apoptosis are extensively focused in areas of research in the context of cancer. The role of cellular autophagy has many biological functions. Autophagy can digest and degrade abnormal proteins and organelles to provide energy and nutrients to hepatocellular carcinoma cells in a state of cellular starvation or stress [7]. Conversely, activation of autophagy can induce apoptosis in hepatocellular carcinoma cells and block the cell cycle, and exert antitumor effects [8]. Additionally, apoptosis is indispensable for the development of the organism. To some extent, apoptosis plays a positive role when the organism is under stress, yet apoptosis plays a negative role in the organism and even assistant tumor resistance when apoptosis is out of control [9, 10].

Some articles indicate that autophagy and apoptosis have intricate relationships that coexist in cells and share a common regulatory element. In the present research, we planned to investigate the effect of CPhGs on apoptosis and autophagy in H22 hepatoma-bearing mice.

## 2. Materials and Methods

### 2.1. Preparation of CPhGs

**Figure 1:**
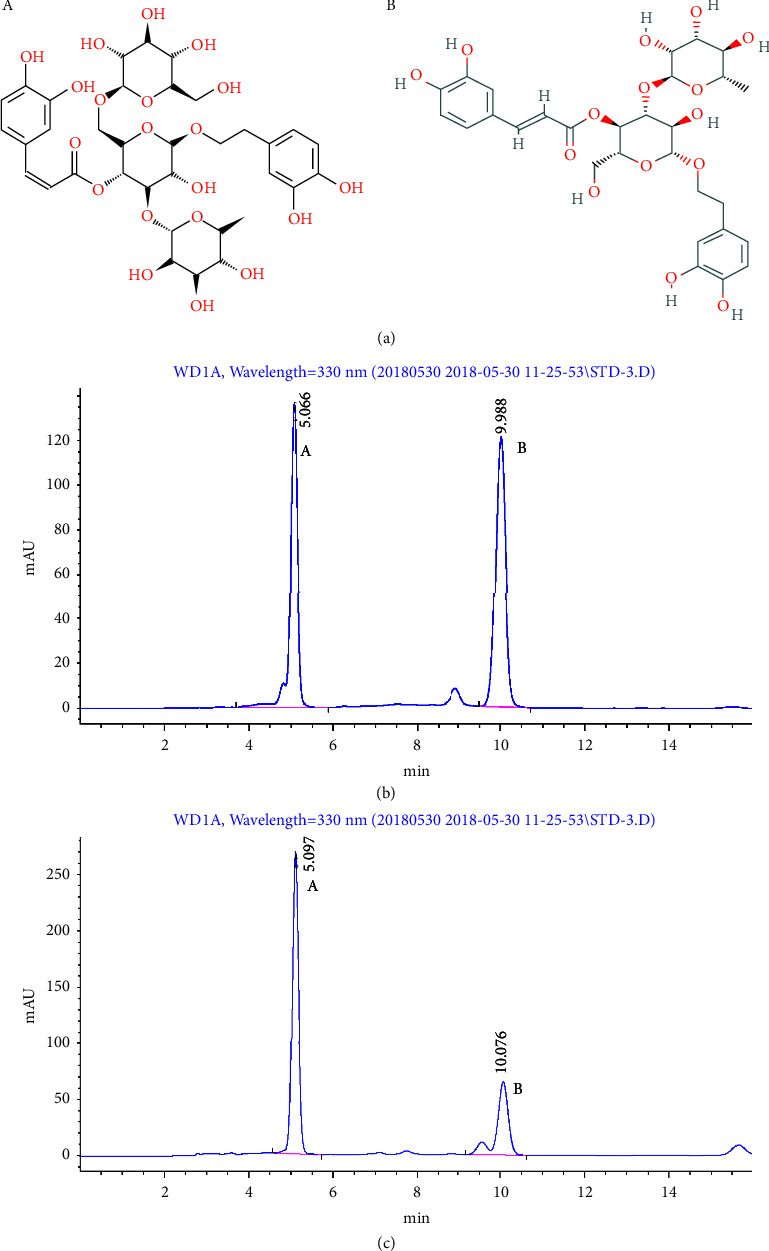
The main components of CPhGs structure and HPLC (a) (A) is the chemical structure diagram of echinacoside, and (B) is the chemical structure diagram of acteoside. (b) Standard sample Atlas, peak A 5.066 min echinacoside, peak B 9.988 min acteoside; (c) The CPhGs extract map used in the experiment, peak A 5.097 min echinacoside, peak B 10.076 min acteoside.

CPhGs were obtained from Hetian DiChen Medical Biotechnology Co. Ltd. (Xinjiang, China). The content of CPhGs was ≥85%. A HPLC method was established to identify the major compounds of CPhGs, including echinacoside (Desite Biotech Co., Ltd., Lot No.: DST200710-038) and acteoside; among them, the echinacoside was more than 40.6%, and that of the acteoside was more than 16.7% (Figure 1).

### 2.2. Cells and Animal

H22 cells were purchased from Shanghai Zhong Qiao Xin Zhou Biotechnology Co., Ltd. (Shanghai, China) and were cultured in 1640-RPMI supplemented with 10% bovine fetal serum (Israeli Biological Industries) and 1% penicillin-streptomycin (Israeli Biological Industries). Cells were grown in a humidified atmosphere containing 5% CO^2^ at 37°C. At 80% confluence, the cells were inoculated into mice. Health Kunming male mice (cleanliness of mice is SPF level, the average body weight of 18–22 g) were purchased from The Animal Experiment Center of Xinjiang Medical University (Animal Production License No., SCXK(Xin) 2018–0002). The feed (Co-60 irradiated) was provided by the Animal Center of Xinjiang Medical University. Animals were raised in individually ventilated cages (IVC, Shandong Xinhua Medical Instrument Co., Ltd., Model: BCR-MI-DZ) and maintained in a room with a controlled temperature (22–24°C), humidity (40–70%), and a 12 h light/dark cycle.

### 2.3. Animal and Experimental Prototype

Kunming mice (male, 4–5 weeks old, weighing 18–20 g) were purchased from the Experimental Animal Center of Xinjiang Medical University (Xinjiang, China) and by the Experimental Animal Ethics Committee of Xinjiang Medical University (license number: IACUC—20201215-16). After one week of acclimatization, the groups were randomized (*n* = 10), except for the control group, and each group was injected with H22 cells (5 × 10^6^ in 200 *μ*L PBS) into the right side of the nude mice. Bodyweight and tumor volume were measured every three days. When 21 days had passed, mice were killed, and tumors were collected, weighed, and imaged. The tumor tissue was snap-frozen in liquid nitrogen and stored at −80°C for subsequent analysis.

### 2.4. Microplate Method

Mouse serum samples were collected and stored in a cryogenic refrigerator (−80°C), and then OD values were measured by the microplate reader (Thermo, USA); all operations are strictly operated according to the kit instructions (C009-2-1 or C010-2-1, Nanjing Jian Cheng Biotechnology, Jiangsu, China). The following was drawing the standard curve and calculating the equation (*R*^2^ ≥ 0.99). Finally, the content of ALT and AST of each sample was calculated according to the standard formula and OD values.

### 2.5. ELisa Assay

The temperature of the ELisa kit and sample needed to recover to room temperature, so we took them out from the refrigerator before the experiment and then quipped them with detergent and reagents according to the manufacturer's instructions. Serum AFP and TNF-content were evaluated using the specific ELISA kit (E-EL-M0049c or E-EL-M2405c, Nanjing Jian Cheng Biotechnology, Jiangsu, China). The detection wavelength was 450 nm, and the standard curve meets the guidelines of *R*^2^ ≥ 0.99.

### 2.6. Histopathological Examination of Tumor

The tumor tissue was fixed with 10% paraformaldehyde for 24 h, embedded in paraffin, and cut into 4 *μ*m thin slices. The nuclei were stained with hematoxylin and 1% alcohol hydrochloric acid, and the cytoplasm was stained with eosin after dewaxing and hydration of sections. Finally, the image was collected under a microscope.

### 2.7. Western Blot

The liver tissue was ground with liquid nitrogen, followed by the extraction of total protein at 4°C. The protein concentration in each sample was measured with a BCA protein concentration determination kit (Thermo Fisher Scientific, US) and adjusted to the concentration with deionized water. The total protein of each group (20 *μ*g) was scattered by sulfate/polyacrylamine gel electrophoresis (SDS-PAGE) and transferred to polyvinylidene difluoride (PVDF) membranes. Next, the PVDF membranes were immersed in fresh, nonfat milk (5%) for 2 h at room temperature and curated with primary antibody for a night in the refrigerator (4°C). The following day, the membrane would be incubated in the secondary antibodies for 2 h at room temperature. Eventually, we visualized these results by ECL with chemiluminescence. In this experiment, the main primary antibodies included cleaved-caspase3 (1 : 1000, Biological Industries bs-0081R), cleaved-PARP (1 : 1000, Biological Industriesbs-55164R), LC3B-II (1 : 500, Biological Industriesbs-8878R), p62 (1 : 2000, Biological Industriesbs-55207R), and secondary antibodies were goat anti-rabbit lgG and goat anti-mouse lgG (1 : 20,000, Proteintech, Wuhan, China). We used *β*-actin (1 : 1000, Biological Industries) as a control.

### 2.8. Immunohistochemistry

The tumor samples were fixed in 10% neutral buffered formalin and embedded in paraffin and were cut into 5 *μ*m thick sections with a microtome, subsequently deparaffinized with xylene, hydrated using a gradient, and subjected to antigen recovery using the citric acid buffer at high temperature. Next, we dropped 3% H2O2 on the slices and incubate them at room temperature for 30 minutes to block endogenous peroxidase [11]. Then, the samples were stained withanti-cleaved-PARP (1 : 300), anti-cleaved-caspase 3 (1 : 200), anti-LC3B-II (1 : 200), and anti-p62 (1 : 300) antibodies at 4°C overnight, and hematoxylin was used for nuclear counterstaining. The nucleus (blue) and the target protein (brown) can be observed under the microscope. The sections were examined by light microscopy and quantification using ImageJ software [12].

### 2.9. TUNEL Apoptosis Assay

The TUNEL assay kit (Solarbio Life Science, T2190) was used according to the manufacturer's instructions. Briefly, paraffin-embedded brain tissue from treated naked mice was dewaxed and dehydrated. After fixing the tissue, permeabilizing the cell membrane, and equilibrating the cells, the slides were incubated with the TUNEL reaction mixture for 2 hours at 37°C in a humidified chamber. Finally, anti-fluorescence quenching was used to connect the seals. Green fluorescence (apoptotic nuclei) can be observed under the fluorescence microscope.

### 2.10. Statistics

All the results were shown as the mean ± standard deviation (SD) from at least three independent experiments. Data were analyzed using SPSS21.0, GraphPad 15.0, and ImageJ1.53t. The variance group was compared by one-way analysis of variance (ANOVA); a significant difference was considered if the *P*value was less than 0.05.

## 3. Results

### 3.1. CPhGs Treatment Reduced Liver Tumorigenesis of H22 Hepatoma-Bearing Mice

**Figure 2:**
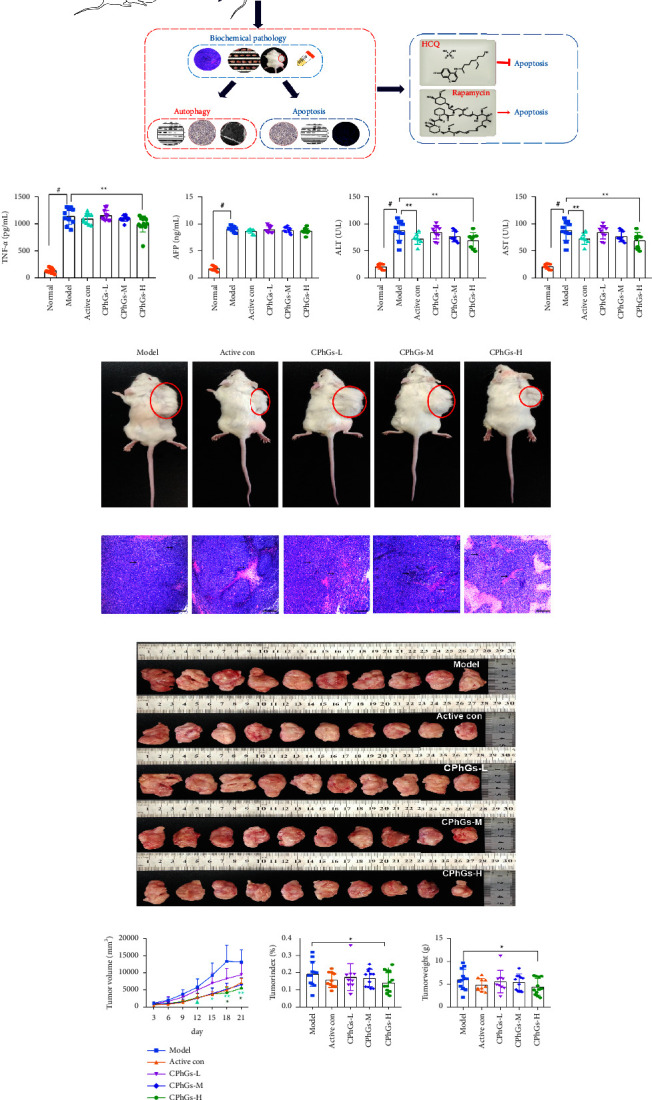
Inhibitory effect of CPhGs on tumor and hepatoprotective effect of H22 tumor-bearing mice. (a) Experimental schema figure. (b) The expression levels of serum TNF-*α*, ALT, AST, and AFP in mice of each intervention group. (c) Comparison of the size of unisolated tumors in each group of mice. (d) Paraffin-embedded sections of tumors from mice were stained with hematoxylin and eosin (scale bar is 50 *μ*m). (e) Comparison of the weight of isolated tumors in each group. (f) The trend graph of tumor growth in each group during the experiment and the comparison of the volume size of isolated tumors in each group. The results were shown as the mean ± SD vs. model group, ^*∗*^*P* < 0.05,^*∗∗*^*P* < 0.01, vs. normal group, ^*#*^*P* < 0.05.

To appraise the anticancer effect of CPhGs *in vivo*, we established an H22 tumor-bearing mice model and observed tumor growth in our experiment (Figure 2(a)). The results are shown in Figure 2(c)–2(f); tumor scale and tumor index decreased significantly in the high-dose CPhGs group, compared with the model group, even less than the positive control group. Notably, in the high-dose CPhGs group, there was a significant abatement after curing for 18 days. Under the microscope, the tumor cells were arranged disorderly and had no organizational structure. There are many cancer cell nests that can be observed. Moreover, the pathological of cancer cells in the low-dose CPhGs group was like the model group, and the fragmented nucleus was observed in the high-dose CPhGs group (Figure 2(d)). To confirm whether the liver function stayed in the injury status in H22 hepatoma-bearing mice, we checked the vital indicators in the serum of liver function. As shown in Figure 2(b), the activities of serum Alanine transaminase (ALT) and Aspartate aminotransferase (AST) were increased in the model group compared with the control group. However, CPhGs treatment in the high-dose group (500 mg/kg) caused a significant promotion. In addition, tumor necrosis factor-alpha (TNF-*α*) exerts essential biological functions in various cancers. In our research, a substantial increase in the activity of TNF-*α* was observed in the model group, whereas TNF-*α* actions were reversed by high-dose of CPhGs (Figure 2(b)). AFP is a crucial regulator of cell proliferation in liver cancer, its expression in the serum of hepatoma-bearing mice was also higher than in the control group, but the presentation was not reduced after CPhGs treatment lasted for 21 days. At this point, there is a need for additional experiments to explore specific changes and reasons. To sum up, CPhGs suppress the size of the tumor body and improve liver function in H22 hepatoma-bearing mice.

### 3.2. CPhGs Treatment Induced Apoptosis of Tumor in H22 Hepatoma-Bearing Mice

**Figure 3:**
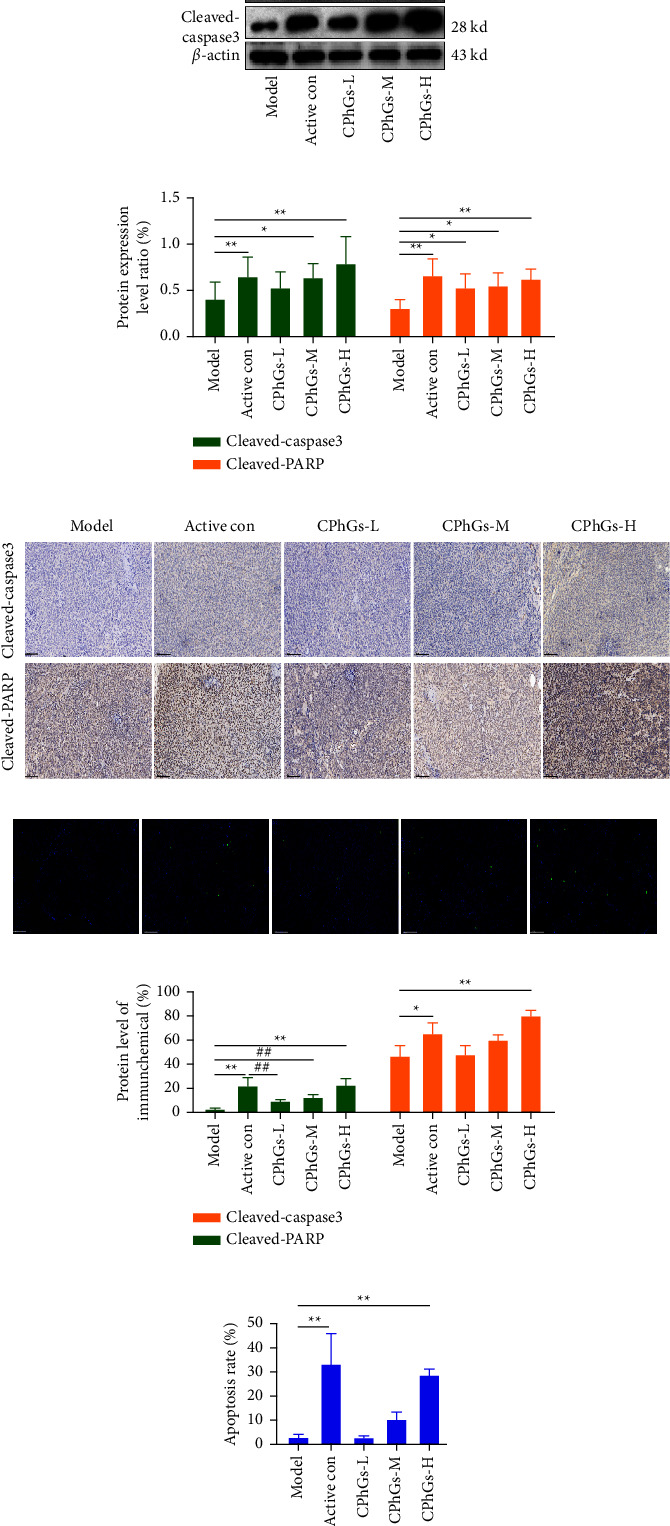
Induction of apoptosis in H22 tumor-bearing mice by CPhGs. (a) Apoptosis-associated protein cleaved caspase-3 and cleaved-PARP detected by western blot. (b) The protein levels of cleaved caspase-3 and cleaved-PARP in tumor issues were calculated using western blot. (c) Cleaved caspase-3 and cleaved-PARP expression of tumors from mice were assayed by IHC (100x, scale bar is 100 *μ*m). (d) TUNEL staining of tumor tissues was used to observe the occurrence of apoptosis in H22 tumor-bearing mice (scale bar is 100 *μ*m). (e) The protein levels of cleaved caspase-3 and cleaved-PARP in tumor issues were calculated depending on IHC. (f) Apoptosis rate of H22 tumor-bearing mice tumor tissue was calculated according to TUNEL staining. The results were shown as the mean ± SD, vs. model group, ^*∗*^*P* < 0.05, ^*∗∗*^*P* < 0.01, vs. normal group, ^#^*P* < 0.05.

We further investigated the effects of CPhGs on the apoptosis of tumor in mice. Using TUNEL to measure cell apoptosis (Figure 3(d) and 3(f)), we found that all groups of CPhGs showed positive TUNEL signals, yet only the CPhGs high-dose group showed an obvious difference compared with the model group. In addition, the activation of apoptosis of the CPhGs high-dose group did as well as the positive control group (Xihuang Wan treatment). Moreover, the results in immunohistochemistry (Figure 3(c) and 3(e)) showed that cleaved-PARP and cleaved-caspase 3 could be located at different places in the cell, cleaved-PARP could only be observed in the nucleus, while cleaved-caspase 3 could appear in both the nucleus and cytoplasm, mainly in the cytoplasm. Besides, we got the help of quantitative and statistical analysis, and it turned out that CPhGs increased the expression of cleaved-PARP and cleaved-caspase3 in a dose-dependent manner. The expression of cleaved-PARP was obviously different between each of the CPhGs-treatment groups and the model group, and the expression of cleaved caspase-3 in the medium and high-dose groups was remarkably higher than that in the model group. Furthermore, the western blot results (Figure 3(a) and 3(b)) showed that CPhGs could increase the expression of cleaved-PARP and cleaved-caspase 3 in a dose-dependent manner. Compared with the model group, the CPhGs high-dose group and the positive control group exhibited a more significant promoting effect on the cleaved-PARP and cleaved-caspase 3, and there were statistically significant differences. Thus, CPhGs could effectively promote the apoptosis of tumors, and the induction effect of CPhGs on apoptosis is consistent with the Xihuang Wan.

### 3.3. CPhGs Treatment-Induced Autophagy of Tumor in H22 Hepatoma-Bearing Mice

**Figure 4:**
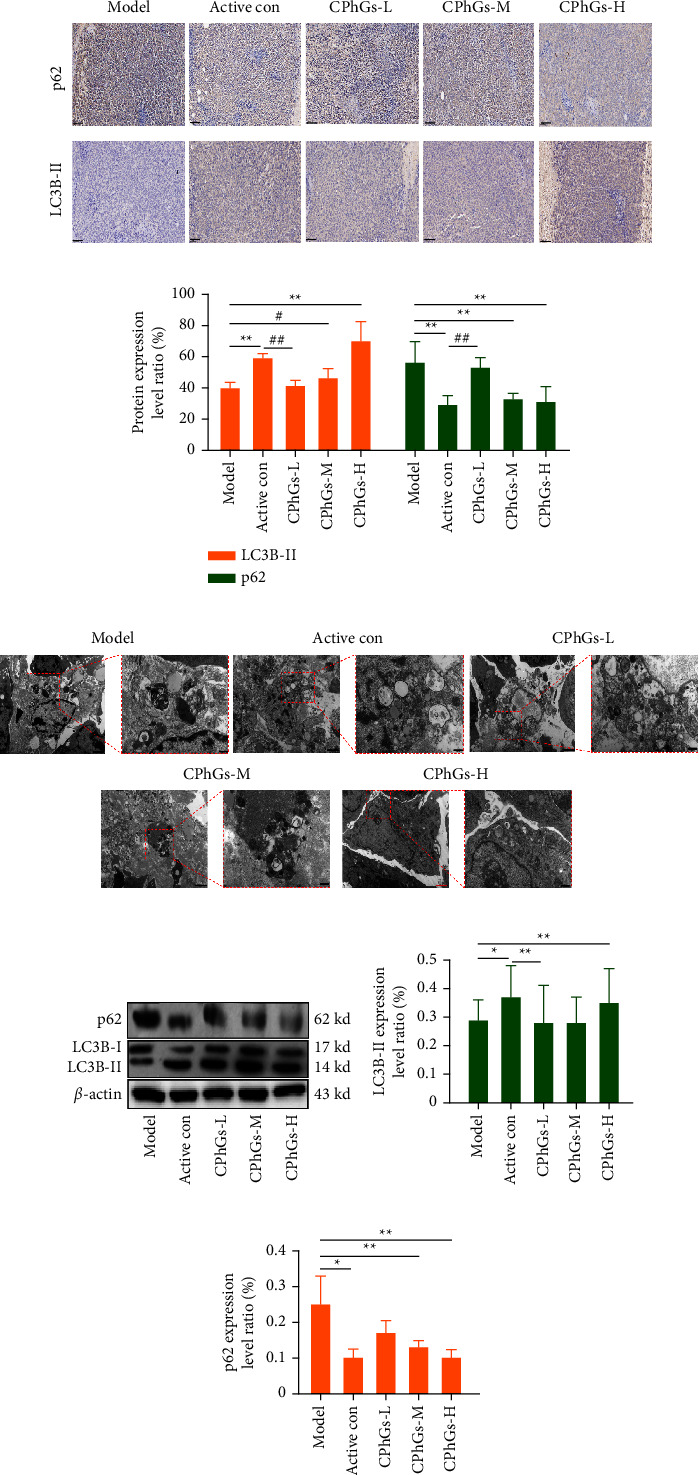
Induction of autophagy in H22 tumor-bearing mice by CPhGs. (a) p62 and LC3B-II expression of tumor from mice were assayed by IHC (100×, scale bar is 100 *μ*m). (b) The protein levels of p62 and LC3B-II in of tumor issue were calculated depending on IHC. (c) Ultrastructure of autophagic vesicles in tumor tissue of H22 tumor-bearing mice by transmission electron microscopy (3000x/10000x, scale bar is 500 nm and 2 *μ*m, respectively). (d) Autophagy-associated protein p62 and LC3B-II detected by western blot. (e, f) p62 and LC3B-II protein levels in tumor tissues were calculated using western blot. The results are shown as the mean ± SD vs. model group, ^*∗*^*P* < 0.05, ^*∗∗*^*P* < 0.01, vs. normal group, ^#^*P* < 0.05.

**Figure 5:**
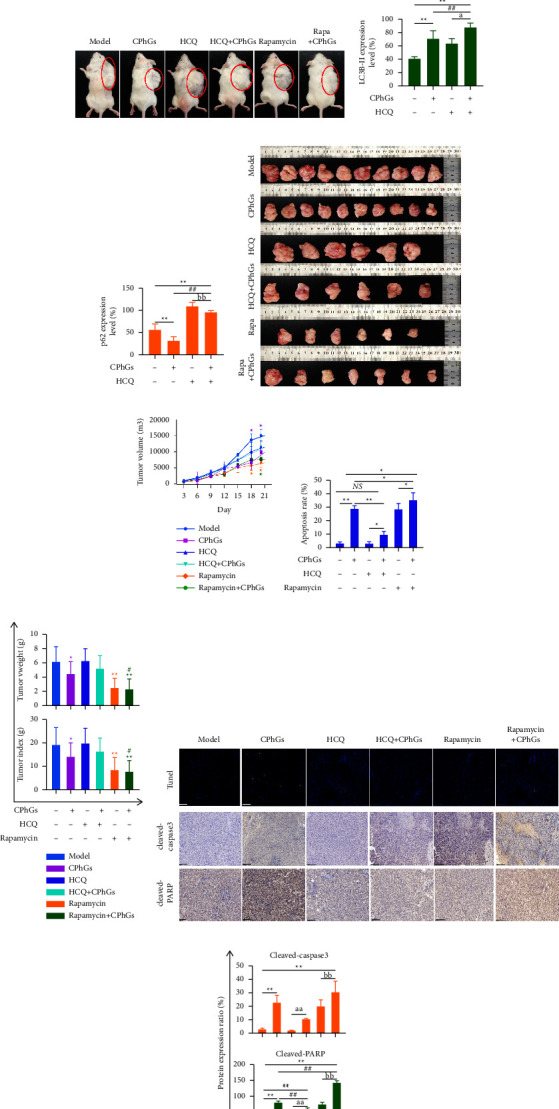
Effect of autophagy regulation on apoptosis induced by CPhGs in H22 tumor-bearing mice. (a) Ultrastructure of autophagic vesicles in tumor tissue of H22 tumor-bearing mice by transmission electron microscopy (3000x/10000x, scale bar is 500 nm and 2 *μ*m, respectively). (b) p62 and LC3B-II expression of tumor from mice were assayed by IHC (100x, scale bar is 100 *μ*m). (c) Comparison of the size of unisolated tumors in each group of mice. (d, e) Protein levels of p62 and LC3B-II in of tumor issue were calculated depending on IHC. (f) Comparison of the size of isolated tumors in each group. (g, i) Trend graph of tumor growth in each group during the experiment and the comparison of the volume size of isolated tumors in each group. (j) Cleaved caspase-3 and cleaved-PARP expression of tumor from mice were assayed by IHC (100x, scale bar is 100 *μ*m) and TUNEL staining of tumor tissues was used to observe the occurrence of apoptosis in tumor cells H22 tumor-bearing mice (scale bar is 100 *μ*m). (h) Apoptosis rate of H22 tumor-bearing mice tumor tissue was calculated according to TUNEL staining. (k) The protein levels of cleaved caspase-3 and cleaved-PARP in of tumor issue were calculated depending on IHC. The results were shown as the mean ± SD vs. model group, ^*∗*^*P* < 0.05,^*∗∗*^*P* < 0.01, vs. CPhGs group, ^*#*^*P* < 0.05, ^*##*^*P* < 0.01 vs. HCQ group, ^*a*^*P* < 0.05, ^*aa*^*P* < 0.01 vs. Rapamycin group, ^*b*^*P* < 0.05, ^*bb*^*P* < 0.01.

To further identify whether CPhGs induced autophagy, we checked autophagy-related indicators. Immunohistochemistry showed (Figure 4(a) and 4(b)) that LC3B-II and p62 could locate in the cytoplasm and CPhGs can increase the expression of LC3B-II and decrease the expression of p62 in a dose-dependent way. Further, compared with the model group, the level of LC3B-II increased significantly in the CPhGs high-dose group, whereas the cell quantity of p62 decreased significantly in the CPhGs high and middle-dose group. The presentation of the western blot is the same as the immunohistochemistry (Figure 4(d)–4(f)). Need to add that, the positive control group showed the same results as the CPhGs high-dose group. Under transmission electron microscopy (Figure 4(c)), we could observe increasing autophagosomes with the treatment of CPhGs. It has been reported that the increase in intracellular autophagic vesicles could be that autophagy is induction or that autophagic flow is blocked [13]. Therefore, we used HCQ, an inhibitor of the autophagic degradation phase, to explore one of these possibilities as interference and intervened simultaneously with CPhGs. TEM (Figure 5(a)) revealed that the combined intervention group of HCQ and CPhGs increased autophagic vesicles more than the CPhGs group. In addition, immunohistochemistry showed that the expression of LC3B-II was increased in the HCQ and CPhGs-alone treatment group, and the expression of LC3B-II in the HCQ + CPhGs group was higher than in the HCQ group, whereas the expression of p62 was opposite.

### 3.4. CPhGs Activates Autophagy as a Facilitating Death Mechanism in H22 Hepatoma-Bearing Mice

Autophagy is two possibilities that determine cell fate; it can promote apoptosis or promote survival in the same cells. Therefore, we further explored the effect of autophagy induced by CPhGs on tumor fate in H22 hepatoma-bearing mice. Tumor size was recorded (Figure5(c), 5(f), 5(g), 5(i)), and the tumor volume, weight, and index decreased significantly in the CPhGs, rapamycin, and rapamycin + CPhGs groups compared with the model group on the 18th and 21st days. Although the tumor volume, weight, and index of the HCQ group were higher than the model group, and CPhGs combined with HCQ intervention decreased it, the changes were not statistically significant. To understand this, we further detected the effect of autophagy inhibitors and inducers on apoptosis (Figure 5(h), 5(j), 5(k)). In the HCQ group, the TUNEL-positive cells had no obvious change compared to the model group, and the expression of cleaved-caspase 3 and cleaved-PARP were decreased, and only the decrease of cleaved-PARP appeared to be statistically significant. In contrast, the opposite phenomenon was observed after rapamycin and CPhGs-alone treatment, the changes were more pronounced in the group of CPhGs combined with rapamycin. In conclusion, we hypothesize that CPhGs induce autophagic death, which affects the level of occurrence of apoptosis in tumor cells when autophagy is blocked.

## 4. Discussion

CPhGs, listed in the Chinese Pharmacopoeia, were only reported to be effective anticancer agents. According to previous experiments, echinacoside could cure liver cancer by inhibiting oncogenes UBR5 [14]. Additionally, CPhGs combined with cisplatin further inhibited the growth of H22 cells and reduced the side effects of cisplatin [15]. However, CPhGs can inhibit liver cancer progression, but its specific mechanism needs to continue to be discussed. To shed light on the underlying molecular mechanisms, we present here novel data showing that CPhGs treatment for 3 weeks in H22 tumor-bearing mice not only inhibited tumor growth but also increased the level of apoptosis and autophagy. CPhGs did not cause weight loss and hepatotoxicity in animals while resisting liver cancer.

Other researchers have found that CPhGs have liver protective effects [16, 17]. Equally, the current study found that the levels of AST and ALT were elevated in H22 tumor-bearing mice, which could be reversed by CPhGs, suggesting that CPhGs exert hepatoprotective effects. Under physiological conditions, AST and ALT are mainly distributed in cells, and the content in serum is low, only in pathological conditions, and the content of AST and ALT in serum will rise [18, 19]. Besides, we found that CPhGs reduced TNF-*α*, while CPhGs lead tumors to liquefy or vanish. TNF-*α* can promote and inhibit tumor growth under intricate conditions [20]. Other researchers found that TNF-*α* could upregulate the expression of caspase 3, an apoptosis-related protein [21]. AFP is a standard marker for the early diagnosis of primary liver cancer in clinics, and AFP expression level negatively correlates with patient survival rate [22]. However, our experiment showed that CPhGs could not decrease the AFP contents after the intervention with CPhGs. Some reports have shown that AFP plays a complicated role in regulating proliferation, apoptosis, autophagy, and inhibit the immune response of cells [23]. In our study, we did not know the sensitivity of AFP indicators, and in other studies of CPhGs antitumor, AFP changes were not mentioned [15, 24]. In conclusion, we still need more detailed experimental protocols to verify the changes and effects of AFP in CPhGs anti-HCC.

It is known that the imbalance between proliferation and apoptosis leads to the infinite expansion of tumors [25]. In this study, CPhGs inhibited tumor growth while inducing apoptosis of tumor cells, and the cleaved-caspase 3 and cleaved-PARP were upregulated after CPhGs treatment. Similarly, Yuan et al. demonstrated that CPhGs significantly activated cleaved caspase-3 and cleaved-PARP of H22 cells *in vitro* [24]. In addition, echinacoside, a representative CPhGs, induced apoptosis via caspase 3 and PARP activation in colostral cancer [26]. Caspase3 together with PARP is an important indicator for apoptosis detection [27]. Caspase3 is generally not enzymatically active until an apoptotic signal emerges that allows it to be cleaved to cleaved caspase-3 [24]. Then cleaved-caspase 3 sheared PARP to guarantee the smooth completion of apoptosis [28]. Li et al. have found that CPhGs induces apoptosis in Eca-109 cells via a mitochondria-dependent pathway; in this process, CPhGs-treatment caused the release of cytochrome c, which promotes the cleavage of caspase3 indirectly, and then cleaves PARP to induce apoptosis [29]. In B16–F10 cells, CPhGs increased the level of CD4^+^ and CD8^+^ T cells and induce the mitochondrial pathway of apoptosis, which suggests that CPhGs may regulate the immunoregulatory effects to inhibit the growth of tumor [30]. The above results suggested that CPhGs may inhibit tumor growth through multiple targets and pathways. However, the present results only show that apoptosis occurred, and the specific apoptotic pathway remains unclear. According to the report, apoptotic pathways include several styles such as cell-intrinsic and -extrinsic processes triggered by cellular stress, DNA damage, and immune surveillance mechanisms [31]. Therefore, we need to make a profound study.

Autophagy is the primary catabolic process in eukaryotic cells to clear the accumulation of misfolded proteins and the damage of organelles, whereas its role in anticancer therapeutic is still controversial [32]. The present data demonstrate that CPhGs significantly increased the activation of LC3B-II, decreased the expression of p62, and promoted the formation of autophagosomes. Autophagy is a highly dynamic process in the cells; therefore, static assays targeting autophagy, such as the detection of LC3B-II and p62 expression, cannot accurately quantify the autophagic flux [13]. So, we need to inhibit the conjunction of autophagosomes and lysosomes with the help of the autophagy inhibitor HCQ to detect the specific status of autophagic flux [33]. Furthermore, we modulated the autophagic degradation phase by HCQ. Compared with HCQ alone, LC3B-II expression increased, while the p62 expression decreased in the HCQ + CPhGs group. Our results found that CPhGs induced autophagy in H22 tumor-bearing mice. In other study, acteoside, a representative CPhGs, could improve cancer-related fatigue by inducing mitophagy in skeletal muscle [34]. Cistanoside A appears to be a potent inducer of autophagy, thereby resulting in enhanced osteogenic differentiation [35]. Therefore, we speculated that CPhGs are autophagy inducers.

However, recent studies have found that autophagy is a double-edged sword [36]. Autophagy may protect cells from death or accelerate cell death in different physiological conditions [37]. Some studies consider that excessive autophagy is a major cause of cell death [38], whereas some studies argue that excessive autophagy reduces apoptosis and promotes cell survival [39]. Analysis of related proteins showed that cleaved caspase-3 and cleaved-PARP were downregulated after treating HCQ, while they were upregulated in the rapamycin group. Besides, the degree of apoptosis was further increased after the intervention with CPhGs. Mamoru et al. found that HCQ effectively promotes Bcl-xlinhibition-induced apoptosis in BxPC-3 human pancreatic cancer cells [40]. Other results found that salidroside could protect the spinal cord neurons after ischemia-reperfusion injury, which may rely on the salidroside to promote autophagy-reducing apoptosis [39]. Unlike our results, most of the research concluded that autophagy inhibition could be an effective approach in advanced cancer, but still there are opposite results [36]. Li et al. found that rapamycin-induced apoptosis in KHE cells [41]. There are many factors and mechanisms responsible for this difference, and the specific apoptotic pathway and the crosstalk between apoptosis and autophagy need to be further explored.

## 5. Conclusion

CPhGs is prescribed to suppress a variety of liver diseases. Our data presented that CPhGs can inhibit HCC via promoting apoptosis and autophagy. There is an interaction between autophagy and apoptosis induced by CPhGs. However, there are some limitations to this research. H22 cells were subcutaneously injected into the flanks of mice, which created ectopic liver cancer. This model cannot mimic the key features of human disease processes. Hence, an orthotopic animal model should be considered to further investigate the precise mechanism of CPhGs in HCC. Clarifying the precise molecular mechanisms underpinning CPhGs-induced autophagy and apoptosis will advance our understanding of CPhGs as a potential therapeutic option for HCC.

## Data Availability

The data used to support the findings of this study are available from the corresponding author upon request.
